# Evolution of Electronic Cigarette Brands From 2013-2014 to 2016-2017: Analysis of Brand Websites

**DOI:** 10.2196/jmir.8550

**Published:** 2018-03-12

**Authors:** Greta Hsu, Jessica Y Sun, Shu-Hong Zhu

**Affiliations:** ^1^ Graduate School of Management University of California Davis Davis, CA United States; ^2^ Moores Cancer Center University of California San Diego La Jolla, CA United States; ^3^ Department of Family Medicine and Public Health University of California San Diego La Jolla, CA United States

**Keywords:** electronic cigarettes, vaping, nicotine, longitudinal studies, internet, market research

## Abstract

**Background:**

The electronic cigarette (e-cigarette) industry has grown in size and organizational complexity in recent years, most notably with the entry of major tobacco companies in 2012 and the proliferation of vape shops. Many brands maintain retail websites that present e-cigarette marketing claims and sell directly to consumers. Understanding of the evolving composition of different types of e-cigarette brand websites is currently underdeveloped.

**Objective:**

This paper presents how e-cigarette brand websites surveyed in 2013-2014 evolved by 2016-2017, and how the websites run by different types of e-cigarette producers currently differ.

**Methods:**

In 2016-2017, we revisited 466 e-cigarette brand websites surveyed in 2013-2014, 288 of which were extant, and identified 145 new English-language websites. We compared product designs, marketing claims, and age-based warnings presented by types of e-cigarette producers: major tobacco companies, independent vape shops, and independent internet-only companies.

**Results:**

Among the 433 websites examined in 2016-2017, 12 were owned by major tobacco companies, 162 operated a physical vape shop, and 259 were internet-only operations. Closed-system product designs were sold by 83% (10/12) of tobacco-owned brands. In comparison, 29.0% (47/162, *P*<.001) of vape shop and 55.2% (143/259, *P*=.06) of internet-only brands sold closed-system designs. Compared with vape shop and internet-only brands, tobacco-owned brands offered a smaller set of product models (*P* values <.001) and a narrower range of flavors (*P* values <.01), with greater emphasis on the traditional combustible cigarette flavors of tobacco and menthol (*P* values <.001). Tobacco-owned brands also offered a narrower range of nicotine options than the vape shops (*P*=.002) and were less likely to offer nicotine-free e-liquid compared with internet-only and vape shop brands (*P* values <.001). Finally, 83% (10/12) of tobacco-owned brand websites featured age verification pop-up windows. In comparison, only 50.2% (130/259) of internet-only brands (*P*=.01) and 60.5% (98/162) of vape shop brands (*P*=.06) featured age verification windows. Websites surveyed in both 2013-2014 and 2016-2017 became more likely to sell open-system mods (*P*<.001) and sold an increased number of product models (*P*<.001), flavors (*P*<.001), and nicotine options (*P*<.001). Prevalence of several types of claims decreased significantly, including indirect claims regarding smoking cessation (*P*<.001), claims regarding e-cigarettes as healthier (*P*<.001), less expensive (*P*<.001), and usable in more places (*P*<.001) compared with combustible cigarettes.

**Conclusions:**

The number of e-cigarette brands has not appeared to increase since 2014, even as website messaging evolved, with brands owned by tobacco companies and vape shops pulling in opposite directions. Brands owned by tobacco companies offered a limited range of e-cigarette products, whereas brands owned by vape shops emphasized a panoply of flavor and nicotine options. Furthermore, the Food and Drug Administration’s regulatory action may influence the types of e-cigarette products offered and the market shares of various companies.

## Introduction

### Background

Since its introduction in the US market in 2007, the electronic nicotine delivery system (ENDS) industry has grown to an estimated US $3.5 billion market [1] with 3 types of electronic cigarette (e-cigarette) products: cigalikes, eGos (or pen-style e-cigarettes), and mods. Cigalikes came on the market first, with slim cylindrical closed-system designs that use prefilled cartridges to maximize the ease of use. eGos and mods are advanced open-system designs that allow users to fill their own e-liquid solution, and they often have adjustable e-liquid heating temperatures, allowing a customized nicotine yield and puff volume [2-5]. eGos have larger cylindrical shapes and stronger batteries than cigalikes, whereas mods are the most customizable and come in a wide range of shapes and sizes.

As the e-cigarette market’s product landscape has evolved, so has its organizational composition. Major tobacco companies such as Lorillard, Altria Group, and Reynolds American began entering the e-cigarette industry in 2012 and have increasingly dominated its market share [6]. Vape shops—independent retail shops that specialize in ENDS products—also appear to have proliferated at a rapid pace [7-9]. Many of these different types of brands maintain retail websites that present marketing claims about e-cigarettes and sell ENDS products directly to consumers. A large proportion of e-cigarette sales are conducted through Web-based channels, which due to the industry’s young and historically unregulated status have not been well tracked [10].

Prior studies conducted by public health researchers reveal important patterns in the advancement of marketing claims. For example, e-cigarette websites often display claims about health or smoking cessation benefits of e-cigarette [11,12], and older e-cigarette brands are more likely to advance claims regarding harm reduction and smoking cessation relative to newer brands [13]. Studies have also found differences in products sold across different types of brands. In 2015, researchers found that major tobacco companies were likely to offer e-cigarette products with closed-system designs and more limited flavors than independents [14].

One large-scale survey documented products sold and claims advanced by 466 e-cigarette websites from December 2013 to January 2014 [13]. We conduct a follow-up to that survey for the period surrounding the finalization of the Food and Drug Administration’s (FDA’s) Deeming Rule in August 2016, which extended the FDA’s regulatory authority under the Family Smoking Prevention and Tobacco Control Act to the ENDS industry [15]. The Deeming Rule has generated a host of varied reactions, ranging from praise for reining in an unregulated product increasingly popular among youths to concern that strict regulatory oversight will limit smokers’ options for an increasingly popular method for smoking cessation [16]. Given that the FDA recently announced plans to review and revise its regulatory oversight rules [17], gaining a better understanding of the products, marketing claims, and age-based protections offered by different types of brands may help regulators better anticipate the impact of regulatory oversight on this young industry’s evolution. Differences along these dimensions are potentially important because they relate to the kinds of products e-cigarette users have access to, the information regarding e-cigarettes that potential and current e-cigarette users are presented with, and the ability of youths to gain access to e-cigarettes.

### Study Question

This study examined all the websites from the 2013-2014 study to determine how many continued to operate in 2016-2017. In addition, we conducted another broad search for e-cigarette brands sold online in 2016-2017. We used the same methodology as the 2013-2014 survey, with particular attention to the ownership of these brands—whether they were owned by major tobacco companies, independent vape shops, or independent internet-only retail companies.

Two main issues were considered. First, did the product designs sold, claims advanced, and age-based protections offered by major tobacco-owned brands, internet-only, and vape shop brands differ during the more recent period? Second, how have brands sampled back in 2013-2014 evolved? For example, did brands change their proclivity to advance claims regarding harm reduction and smoking cessation? Prior studies have conducted surveys of e-cigarette brand websites at single points in time [11,12,14,18], and one study sampled the industry for 2 consecutive years [19]. However, we are not aware of studies tracking the same set of websites over time. General patterns in these changes may provide some indication of how industry incumbents will continue to evolve as this young industry develops. After documenting e-cigarette brands sold on the internet, we consider the potential public health and regulatory implications of our findings. Although we cannot definitively determine how regulatory oversight will shape this young market, our findings suggest potential issues that regulators should consider going forward.

## Methods

### Internet Search

To create our dataset, we first searched the list of websites surveyed in 2013-2014 [13] to identify those still conducting online retail operations as of July 2016. To this dataset, we added websites based on a new internet search of e-cigarette brands, conducted also in July 2016. This search mirrored the structure of the earlier surveys. Using 3 search engines (Google, Yahoo, and Bing), we searched for e-cigarette brands using the following keywords: e-cigarette, e cigarette, e-cig, e cig, ecig, ecigs, electronic cigarette, electronic cig, electronic nicotine delivery system, vape, vaper, and vaping. A website was included as an e-cigarette brand website if it sold e-cigarette hardware and identified at least one hardware or e-liquid product as its own. We included all e-cigarette brand websites listed on the first 30 pages of each search, excluding the following: non-English websites; websites that did not sell products directly to the general public (eg, product review sites, manufacturer sites); websites selling cannabis-only products; and resale sites (eg, Amazon, eBay).

### Brands and Models

Through a 2016 US Department of Health and Human Services industry report, organizational information listed on brand websites, and publicly available information on the internet (eg, company press releases), we identified brands owned by major tobacco companies [6]. Among the remaining brands, we distinguished between those that operated their own physical vape shop versus internet-only operations. Brands were treated as operating a physical vape shop if they ran a retail brick-and-mortar store selling ENDS products. Brands that only operated at mall kiosks and at locations that only allow for pickup of online sales were not considered as vape shops.

From August 2016 to February 2017, a project manager and 5 trained research assistants coded the websites for age-based access restrictions, product characteristics, product claims, e-liquid flavors, ingredients, and nicotine strengths. Coding was based on the 2013-2014 survey codebook. To ensure consistency with the 2013-2014 coding, the project manager overseeing the earlier survey provided training and extensive consultation to the project manager for the 2016-2017 survey. When training the research assistants, an initial set of websites were coded by each of the research assistants to identify and resolve any discrepancies in coding approaches, with extensive feedback given to ensure consistency in approaches. To further ensure consistency, regular checks of the website codes were conducted by the project manager throughout the coding process.

Each e-cigarette sold through a brand website was coded as cigalike, eGo, or mod. Every distinct e-cigarette model sold, including those of competitors’ brands sold on a brand’s website, were coded. The e-cigarettes that only varied in color or flavor of e-liquid were not counted as separate models.

### Claims and Disclaimers About Electronic Cigarettes

Research assistants reviewed entire websites to determine the presence or absence of several types of claims and disclaimers. The following smoking cessation–related statements were coded: (1) a direct claim of e-cigarettes as an effective quitting aid; (2) an indirect claim (eg, a featured customer testimonial) of e-cigarettes as an effective quitting aid; and (3) a disclaimer that e-cigarettes are not approved as smoking cessation devices. We also coded whether websites claimed e-cigarettes are healthier/safer in comparison with combustible cigarettes. Additionally, we coded for claims regarding social benefits, including that e-cigarettes (1) are less expensive, (2) can be smoked in more places, (3) are cleaner or less messy/smelly, and (4) are more socially accepted when compared with combustible cigarettes. Age-related disclaimers were also coded, including a disclaimer that e-cigarettes are not intended for youths/minors and the presence of a pop-up window that asks website visitors to self-report either their age or whether they meet a minimum age threshold.

### Flavors

The research assistants recorded whether the websites sold e-liquid (in either prefilled or liquid bottle formats), and they also recorded every distinct flavor of e-cigarette sold by each website. Distinct flavors were indicated by distinct linguistic labels for flavor (eg, “Cinnamon” and “Red Hot Cinnamon” were treated as distinct flavors). A flavor label did not include the brand names—for example, Brand X’s “Cinnamon” and Brand Y’s “Cinnamon” were treated as the same flavor. The main flavors coded were as follows: tobacco, menthol, alcohol/drinks, fruit, and dessert/candy. About 4.5% of flavors did not fall into these main categories. The flavors were generally coded by their first ingredient, with 2 exceptions: flavors that referenced tobacco were coded as tobacco and those described as minty, icy, or frosty were coded as menthol. Do-it-yourself flavor concentrates were excluded from this coding. We studied the proportion of the total flavors sold per brand in each of the main flavor categories.

### Nicotine Strengths

For each website, research assistants recorded all distinct nicotine strengths listed. We reported the number of distinct strengths sold as well as whether each website offered zero-nicotine or nicotine-free options.

### Statistical Analysis

Chi-square tests were conducted to determine significant differences in the types of product models sold, claims advanced by different brand types, and differences in flavor types sold. The McNemar test for paired data was used to examine changes in brands’ product model, claims, and flavors sold from 2013-204 to 2016-2017. Moreover, *t* tests were used to examine differences in mean product model count and mean distinct flavor count. STATA version 14.2 (StataCorp LLC) was used for all analyses. Since 3 separate tests were performed (to compare tobacco-owned, internet-only, and vape shops to each other), we used a *P* value of .01 instead of the more conventional .05 for determining statistical significance.

## Results

### Brands and Models

A total of 178 (38.2%) of the 466 brands included in the 2013-2014 survey were no longer in operation as of July 2016, indicating substantial attrition in websites from 2013-2014 to 2016-2017. The 288 still operating in 2016-2017 provide a subset for longitudinal comparison. A total of 145 additional brands were uncovered through the 2016-2017 internet search. In total, we coded 433 websites in the 2016-2017 survey. We determined that 12 brands were owned by major tobacco companies, including *Blu* (Imperial Tobacco), *Logic* (Japan Tobacco), *MarkTen* (Altria Group, Inc.), and *VUSE* (Reynolds American, Inc.). Among the remaining brands, 162 operated their own physical vape shop and 259 were internet-only operations.

**Table 1:** A comparison of products and models offered by e-cigarette brands.

Type	2016-2017 survey (433 brands)						Evolution over time (288 brands)		
	Major tobacco (N=12), n (%)	Internet only (N=259), n (%)	Vape shop (N=162), n (%)	Tobacco vs internet, *P* value^a^	Tobacco vs vape shop, *P* value	Internet vs ape shop, *P* value	2013-2014 survey (N=288), n (%)	2016-2017 survey (N=288), n (%)	*P* value
Cigalike	10 (83.3)	143 (55.2)	47 (29.0)	.06	<.001	<.001	202 (70.1)	155 (53.8)	<.001
eGo	7 (58.3)	198 (76.4)	132 (81.5)	.15	.05	.22	208 (72.2)	227 (78.8)	.03
Mod	2 (16.7)	155 (59.8)	151 (93.2)	.003	<.001	<.001	117 (40.6)	190 (66.0)	<.001
Number of models, mean^b^	2.8	12.3	30.0	.08	<.001	<.001	6.7	17.2	<.001

^a^Comparisons between percentages were calculated using chi-square analysis in columns 1-6 and using McNemar test for paired data in columns 7-9.

^b^Comparisons between means were calculated with two-tailed *t* tests.

Table 1 shows data on product models sold online in 2016-2017. The first 3 columns list the percentage of brands in each type that sold cigalike, eGos, and mod designs through their websites. Columns 4-6 show *P* values for differences between brand types.

Cigalikes were sold by 83.3% (10/12) of the major tobacco company-owned brands compared with 55.2% of internet-only brands (143/259, *P*=.06) and 29.0% of vape shop brands (47/162, *P*<.001). Meanwhile, 16.7% (2/12) of tobacco-owned brands sold mods. This is significantly lower than the internet brands (59.8%, 155/259, *P*<.001) and vape shop brands (93.2%, 151/162, *P*<.001). Tobacco brands sold fewer product models on average (2.8) compared with internet (12.3, *P*=.08) and vape shop brands (30.0; *P*<.001). Overall, tobacco-owned brands and vape shop brands show the widest differences in products and models offered.

The last 3 columns of Table 1 compare the change in product types sold in 2013-2014 versus 2016-2017 among the 288 brands captured in both surveys. Brands became less likely to sell cigalikes (*P*<.001) and more likely to sell mods (*P*<.001) by 2016-2017. They also significantly increased their average number of product models sold from 6.7 to 17.2 (*P*<.001).

### Claims and Disclaimers About Electronic Cigarettes

The columns 1-3 in Table 2 compare marketing claims made by different types of brand websites in 2016-2017. Overall, a low proportion of brands advanced direct claims regarding e-cigarettes as a method for smoking cessation, and none of the 12 tobacco-owned brands did so. Brands were more likely to advance indirect than direct claims. There were no significant differences between tobacco-owned brands versus vape shop and internet-only brands in terms of proclivity to advance smoking cessation claims, health-related claims, or social claims. Between internet and vape shop brands, the former were more likely to advance the social claims of e-cigarettes as being allowed in more places (*P*<.001) and being cleaner (*P*<.001) when compared with combustible cigarettes.

The majority of brands advanced disclaimers regarding e-cigarettes as not intended for youths/minors. Tobacco company-owned brands and internet-only brands show the greatest difference here. Moreover, 83.3% (10/12) of the tobacco-owned brands had an age-based pop-up window—a higher proportion than internet-only brands (50.2%, 130/259, *P*=.01). This proportion was also higher than the vape shop brands (60.5%, 98/162), but not statistically significant (*P*=.06).

The last 3 columns of Table 2 examine change in websites’ claims from 2013-2014 to 2016-2017. Of the 6 claims coded in both surveys, 4 showed significant change. Although 57.3% (165/288) of brands advanced indirect smoking cessation claims in 2013-2014, less than half of this percentage (21.9%, 63/288) advanced them in 2016-2017 (*P*<.001). Brands also became significantly less likely to advance claims that e-cigarettes were healthier (*P*<.001), less expensive (*P*<.001), and could be smoked in more places (*P*<.001) than combustible cigarettes.

### Flavors

The total count of distinct flavors sold by websites studied in 2016-2017 was 15,586—more than double the 7764 flavor labels found in 2013-2014. Major tobacco companies were less likely to sell e-liquids (66.7%, 8/12 sold e-liquids) relative to the internet-only (86.9%, 225/259, *P*=.05) and vape shop brands (98.1%, 159/162, *P*<.001). Table 3 (columns 1-3) compares flavors sold in 2016-2017 by brand type. Major tobacco companies sold fewer flavors on average (20.7 flavors) through their websites relative to vape shop brands (137.5 flavors, *P*=.002). Tobacco-owned companies sold significantly higher mean proportions of tobacco and menthol flavors relative to the internet and vape shop brands (all *P* values <.001). Conversely, tobacco-owned brands sold significantly lower proportions of alcohol/drink, fruit, and dessert/candy flavors relative to vape shop brands (all *P* values <.01).

Brands present in both survey periods became more likely to sell e-liquids (or prefilled cartridges)—89.6% (258/288) sold e-liquids (or prefilled cartridges) in 2016-2017 compared to 75.7% (218/288) in 2013-2014 (*P*<.001). The last 3 columns of Table 3 show other changes in the brands present in both survey periods. From 2013-2014 to 2016-2017, brands significantly decreased their proportions of tobacco (*P*<.001), menthol (*P*=.009), and alcohol/drink flavors (*P*=.003), and significantly increased their proportion of dessert/candy (*P*<.001) flavors. They also increased their average count of distinct flavors sold—from 49.2 to 81.6 (*P*<.001).

**Table 2:** A comparison of claims and disclaimers made by e-cigarette brands.

Claim		2016-2017 survey (433 brands)^a^						Evolution over time (288 brands)^b^		
		Major tobacco (N=12), n (%)	Internet only (N=259), n (%)	Vape shop (N=162), n (%)	Tobacco vs internet, *P* value	Tobacco vs vape shop, *P* value	Internet vs vape shop, *P* value	2013-2014 survey (N=288), n (%)	2016-2017 survey (N=288), n (%)	*P* value
**Smoking cessation claims**										
	Direct: help quit	0 (0.0)	19 (7.3)	14 (8.6)	.33	.29	.63	29 (10.1)	32 (11.1)	.74
	Indirect: help quit	2 (16.7)	50 (19.3)	23 (14.2)	.82	.81	.18	165 (57.3)	63 (21.9)	<.001
	Not smoking cessation device	5 (41.7)	160 (61.8)	89 (54.9)	.16	.37	.17	160 (55.6)	177 (61.5)	.07
**Health claims**										
	Healthier than smoking	5 (41.7)	129 (49.8)	64 (39.5)	.58	.88	.04	201 (69.8)	151 (52.4)	<.001
**Social claims**										
	Less expensive than smoking	2 (16.7)	116 (44.8)	52 (32.1)	.06	.27	.01	178 (61.8)	125 (43.4)	<.001
	Used in more places	6 (50.0)	119 (45.9)	42 (25.9)	.78	.07	<.001	174 (60.4)	132 (45.8)	<.001
	Cleaner than smoking	3 (25.0)	132 (51.0)	54 (33.3)	.08	.55	<.001	NA^c^	NA	NA
	Socially accepted	1 (8.3)	32 (12.4)	11 (6.8)	.68	.84	.07	NA	NA	NA
**Age claims**										
	Disclaimer: not for minors	11 (91.7)	202 (78.0)	125 (77.2)	.26	.24	.84	NA	NA	NA
	Age pop-up window	10 (83.3)	130 (50.2)	98 (60.5)	.01	.06	.02	NA	NA	NA

^a^Comparison in columns 1-6 were calculated using chi-square analysis.

^b^Comparisons in columns 7-9 were calculated using McNemar test for paired data.

^c^NA: Not available. Since variable was not coded for in the 2013-2014 survey, a longitudinal comparison is not possible.

**Table 3:** Flavors offered by e-cigarette brands.

Variable		2016-2017 survey (417 brands)						Evolution over time (278 brands)		
		Major tobacco (N=10)	Internet only (N=245)	Vape shop (N=162)	Tobacco vs internet, *P* value	Tobacco vs vape shop, *P* value	Internet vs vape shop, *P* value	2013-2014 survey (N=278)	2016-2017 survey (N=278)	*P* value
Number of flavors per brand, mean		20.7	56.3	137.5	.04	.002	<.001	49.2	81.6	<.001
**Mean proportion of total flavors per brand, %^a^**											
	Tobacco	33.2	19.1	11.3	.005	<.001	<.001	21.9	18.1	<.001
	Menthol	27.2	12.9	9.3	<.001	<.001	<.001	13.9	12.4	.009
	Alcohol/Drink	7.6	12.5	12.6	.05	.001	.45	13.9	12.3	.003
	Fruit	18.2	29.1	27.7	.01	<.001	.14	28.0	29.0	.13
	Dessert/Candy	12.1	21.4	35.0	.03	<.001	<.001	16.7	23.9	<.001

^a^Comparisons between means and proportions were calculated with two-tailed *t* tests.

**Table 4:** Nicotine options offered by e-cigarette brands.

Number of nicotine options per brand	2016-2017 survey (407 brands)						Evolution over time (273 brands)		
	Major tobacco (N=10)	Internet only (N=237)	Vape shop (N=160)	Tobacco vs internet, *P* value	Tobacco vs vape shop, *P* value	Internet vs vape shop, *P* value	2013-2014 survey (N=273)	2016-2017 survey (N=273)	*P* value
Mean^a^	4.6	5.9	7.2	.09	.006	<.001	4.9	6.3	<.001
Offers zero nicotine^b^, n (%)	5 (50.0)	211 (89.0)	156 (97.5)	<.001	<.001	.002	248 (90.8)	252 (92.3)	.57

^a^Comparisons between means were calculated with two-tailed *t* tests.

^b^Comparisons between percentages were calculated using chi-square analysis in columns 1-6 and using McNemar test for paired data in columns 7-9.

### Nicotine Strengths

Table 4 shows that, in 2016-2017, tobacco company-owned brands offered fewer nicotine options on average relative to the vape shop brands (*P*=.006) and were less likely to offer zero-nicotine options compared with both vape shops and internet-only brands (*P* values <.001). Almost all the vape shop brands (97.5%, 156/160) and 89.0% (211/237) of internet brands offered a zero-nicotine option, whereas only half of the tobacco-owned brands (50.0%) offered one. The last 3 columns show that, from 2013-2014 to 2016-2017, brands significantly increased their average number of different nicotine options (*P*<.001). There was no change in the likelihood of a zero-nicotine option.

## Discussion

### Principal Findings

The analyses presented here show that major tobacco company-owned e-cigarette brands and smaller, independent e-cigarette brands offered very different portfolios of products through their online retail websites. Vape shop and tobacco company-owned brands appeared the most distinct from one another along a number of dimensions. Compared with vape shop brands, tobacco-owned brands were more likely to sell closed-system designs, were less likely to offer e-liquids, and tended to offer a narrower range of e-liquid flavors with greater emphasis on traditional (ie, tobacco and menthol) flavors. These are all product characteristics that resemble combustible cigarettes in appearance and taste. Vape shop brands, in comparison, were more likely to sell open-system models, focused more on nontraditional e-liquid flavors such as fruit and dessert/candy, offered a greater range of nicotine options, and were more likely to offer nicotine-free e-liquid. Internet-only brands, generally, resembled vape shop brands in their product offerings, although their differences with major tobacco brands were, generally, smaller in magnitude and less statistically significant with regard to product models, flavors, and nicotine options.

These patterns are consistent with research that suggests vape shops focus primarily on newer generation devices and encourage users’ experimentation with a variety of nicotine and e-liquid options [20-22]. Studies also indicate vape shop employees frequently characterize e-cigarettes as smoking cessation devices and, despite lack of formal training, provide counsel regarding smoking cessation to customers [8,23]. Although we found a slightly higher rate of vape shops that claimed direct smoking cessation benefits to e-cigarettes relative to major tobacco brands, this difference was not statistically significant. Given the proliferation of vape shops in the United States, further study of this channel is important to understand the changing economic, social, and cultural dynamics of the e-cigarette market.

Among the 288 brand websites studied in both the 2013-2014 and 2016-2017, we found change in the propensity of e-cigarette brands to advance several types of claims about e-cigarettes. In 2013-2014, a majority of the websites presented indirect smoking cessation claims (57.3%, 165/288). This decreased to 21.9% (63/288) in 2016-2017. We also found significant decreases in claims regarding health and social benefits of e-cigarettes relative to combustible cigarettes. E-cigarette brands appear to have become more conservative in their marketing claims over time.

We also found, through our comparison of brands covered in both the 2013-2014 and 2016-2017 surveys, that brands have generally evolved from closed to open product designs, from traditional to nontraditional e-liquid flavors, and toward greater variety in models, flavors, and nicotine options. These general trends are important to keep in mind when considering the potential consequences of the 2016 Deeming Rule, which currently requires e-cigarette manufacturers to complete an application process that includes detailed ingredient, manufacturing, and product labeling/marketing information [24,25]. The FDA estimated that the resources to complete applications for new tobacco products will be considerable, costing between US $117,000 and US $466,000 per product (flavor-strength combination) [26].

A number of researchers and public health officials have raised concern that strict regulatory oversight will suppress product innovation; push out smaller, independent companies with limited resources; and ultimately strengthen market dominance for major tobacco companies [25,27]. Major tobacco companies, whose practices have been shaped by decades of experience with federal regulators, appear well-positioned and well-resourced to gain advantage within a strict regulatory environment. There is also some evidence suggesting that independent brands have already started reducing their product inventory and closing down operations in response to impending FDA oversight [28,29]. Our brand surveys indicate substantial attrition in e-cigarette brands over the past few years. This contrasts sharply with the strong growth in e-cigarette brand websites found by Zhu et al from 2012 to 2014 [13], and it suggests that the industry may be headed toward even greater contraction in the number of brands in the coming years.

Some of the concerns regarding how regulatory oversight will affect the market landscape appear driven by tobacco companies’ history of deceptive business practices and potential for heavily resourced, sophisticated marketing campaigns [11,30]. Our analyses suggest an additional reason for concern. Tobacco-owned brands are more likely to offer a limited range of product designs and flavors that closely resemble the experience of smoking combustible cigarettes. Although science regarding the harm reduction impact of e-cigarettes is still developing, some studies suggest that smokers of cigalikes and traditional tobacco/menthol flavors may be less likely to quit smoking and more likely to remain dual users for prolonged periods of time, which ultimately may be worse for their health outcomes [31,32]. There is also evidence that use of open-system e-cigarette models is associated with higher rates of smoking cessation relative to closed-system models [4,33,34]. This raises the possibility that greater dominance by major tobacco-owned brands and the exit of smaller, independent brands might ultimately limit smokers’ access to e-cigarette models associated with higher quit rates.

On the other hand, we find major tobacco brands are more likely than internet brands to feature a pop-up window asking users to verify their age—83% (10/12) of tobacco brands compared with 50.2% (130/259) of internet and 60.5% (98/162) of vape shop brands. The latter 2 percentages are roughly comparable to recent content analyses of the internet e-cigarette vendors that studied age self-verification practices [18,19]. Of course, this represents a very weak form of age verification, and studies suggest the use of more effective verification methods, such as requiring a driving license number, which is relatively rare [19]. Still, these differences between major tobacco, internet-only, and vape shop brands suggest that major tobacco-owned brands may be more likely than small, independent brands to institute processes preventing online sales to minors. This raises the possibility that the exit of smaller brands could decrease youth access to e-cigarettes. Furthermore, tobacco-owned brands may also be less appealing to youths, as they offered limited ranges of flavors focused on tobacco and menthol, and studies suggest younger smokers find nontobacco flavors, such as fruit and dessert, appealing [35,36].

### Limitations

One limitation of this study is that the search was limited to the first 30 pages of each keyword search. Other e-cigarette brands exist that are not in the database; thus, the total number of e-cigarette brands is likely larger than that reported here [37]. Another limitation is that we were only able to find limited information on tobacco industry ownership of e-cigarette brands. There may be other companies that we are unaware of that are tobacco-industry owned. Finally, as our study focuses on brands’ websites, brands with physical retail stores could present different products and claims through their stores than those captured in this study.

### Conclusions

The FDA has announced its intention to develop ENDS industry regulations that focus on nicotine and promote harm reduction through innovation [17,38]. As regulators consider how to best revise and implement oversight, there is a complex set of issues to take into account. Policies should be designed to discourage youths from starting e-cigarettes and exposing themselves to any potentially negative health effects of nicotine [39,40]. Currently, regulatory requirements do not provide clear guidance regarding specific processes needed to effectively prevent sales to minors [19]. In the 2016-2017 period, internet-only and vape shops brands appeared to take weaker actions to restrict youth access relative to major tobacco-owned brands. At the same time, enough preliminary evidence of a positive association between e-cigarette use and smoking cessation at the population level exists [31,41,42] that the FDA should consider how to encourage companies to continue to develop and offer a range of products. Regulations that involve intensive time- and resource-investments are likely to impose a disproportionate burden on small, independent organizations that have played a key role in developing and offering open-systems models that have been associated with higher quit rates [4,33,34]. Attention should be given to encouraging responsible business practices—particularly with regard to youth access regulation—while streamlining product application requirements to encourage product diversity and innovation among a variety of industry players.

## Abbreviations

e-cigarette: electronic cigarette
ENDS: electronic nicotine delivery system
FDA: Food and Drug Administration
